# Correction: The Functional Influences of Common ABCB1 Genetic Variants on the Inhibition of P-glycoprotein by *Antrodia cinnamomea* Extracts

**DOI:** 10.1371/journal.pone.0116132

**Published:** 2014-12-19

**Authors:** The PLOS ONE Staff

## Notice of Republication

This article was republished on November 19, 2014, due to the release of confidential or copyrighted information. Please view the article again to download the corrected version.
